# Case Report: Trauma group therapy with karate-do for war-traumatized children and adolescents

**DOI:** 10.3389/fpsyg.2024.1301671

**Published:** 2024-10-16

**Authors:** Mirjam Straub Ortiz Montenegro, Patricio Ortiz Montenegro, Fabian Voegeli

**Affiliations:** ^1^School Psychological Service of the City of Zurich, Zurich, Switzerland; ^2^Shudokan Internacional and School Psychological Service of the City of Zurich, Zurich, Switzerland; ^3^Social and Political Thought, York University, Toronto, ON, Canada

**Keywords:** traumatization, migration, man-made trauma, group therapy, Karate-do, self-defense

## Abstract

**Background:**

From the viewpoint of health and education, traumatized children and adolescents who have fled from war and conflict zones to Switzerland represent a high-risk group, as they suffer from psychiatric symptoms to an above-average extent and on several levels: somatic, psychological, psychosomatic, and psychosocial.

**Objectives:**

The complexity and severity of these problems overwhelm the existing school structures in many cases: There is a clear need for psychotherapeutic interventions here that goes beyond purely verbal conversational therapy and provides an holistic concept.

**Methods:**

We propose the following novel approach: “Trauma group therapy with karate-do for war-traumatized children and adolescents” which integrates and applies the evidence-based methods of integrative Budo-Therapy, trauma-focused Cognitive Behavioral Therapy (TF-CBT), Narrative Exposure Therapy (NET) and Integrative Gestalt Therapy according to Dr. Hilarion Petzold (EAG-FPI) and validated it in a group of approximately 12 children from war and conflict zones who attend the public schools of the city of Zürich.

**Results:**

Qualitative feedback received from the teachers is promising. They report that it is now better possible for the children who go to ouer “Trauma group therapy with karate-do for war-traumatized children and adolescents” to concentrate at school and also to better regulate their feelings.

**Conclusion:**

Ouer approach seems to be a promising intervention for traumatized children and adolecents. Though it needs further evaluation.

## 1 Introduction and background

### 1.1 Current situation of mental health of traumatized children and adolescents with a refugee background

War and flight often force children and adolescents and their families to leave everything in their home country, and plunges them into a completely new situation in the country of arrival. According to current figures, 100 million people worldwide are currently on the run, about 42% of them are children and young people (UN Refugee Agency [UNHCR], 2023).

Turrini et al. (2017) carried out an overview article about the mental health of asylum seekers and refugees and found: Although there was substantial variability in prevalence rates, we found that depression and anxiety were at least as frequent as post-traumatic stress disorder, accounting for up to 40% of asylum seekers and refugees. In terms of psychosocial interventions, cognitive behavioral interventions, in particular narrative exposure therapy, were the most studied interventions with positive outcomes against inactive but not active comparators. This outcomes contains one study on Children, with depression and with PTSD (Bronstein and Montgomery, 2011) and one mixed sample (Lindert et al., 2009).

Fazel et al. (2012) conducted a systematic search and review of the evidence-base for individual, family, community, and societal risk and protective factors for the mental health outcomes of children and adolescents of a population of forcibly displaced refugees in both internally displaced and refugee populations (2012).

Their findings were (among others) that many different factors affect the mental health of forcibly displaced children in the presence of substantial life challenges. One we would like to highlighted here is that postmigration factors provide opportunities for high-income countries to intervene directly to achieve improved outcomes for vulnerable children.

### 1.2 Current situation in Switzerland of traumatized children and adolescents with a refugee background

According to the State Secretariat for Migration SEM (2024), 24,511 people applied for asylum in Switzerland in 2022 alone. Of these, 5,695 were children and adolescents under the age of 19 (SEM, asylum statistics, 2022). The number of asylum applications from unaccompanied young people has more than doubled in 2022 (compared to 2021) and has increased fivefold since 2020. In addition, 74,959 people, over a third of whom were minors, applied for protection status in the context of the Ukraine crisis (Save the Children, 2023).

In the current year 2023, 17,358 new asylum applications have already been submitted (SEM, asylum statistics, retrieved on 31.08.2023).

We do not have concrete figures on how many of these refugee children and adolescents are struggling with mental health problems in Switzerland. However, the Swiss Red Cross recently noted in a newspaper article that at the moment, out of about 200,000 people who have applied for asylum in Switzerland in the past, 40–50% are struggling with mental health problems due to trauma (Schmid and Leu, 2023). These percentages also include children and adolescents who have fled to Switzerland together with their parents as well as unaccompanied minors.

Fazel et al. (2012) make the statement, that: “Successful intervention with distressed refugee children requires not only psychotherapeutic skills, but also these in combination with structural interventions.” (2012). As such, the intervention of the School Psychological Service of the City of Zurich can be considered. We regard our group therapy as such a direct, protective intervention for refugee children in Switzerland.

### 1.3 Traumatized children and adolescents with a refugee background in the school system

In Switzerland, children and adolescents of school age are compulsorily enrolled in elementary school. According to the Elementary School Act of the canton of Kanton Zürich (2005), the support of traumatized children within a protected framework of group therapy with the aim that they then can get better involved in school is an important goal.

Like Lic. Phil. Kohli (2021), the head of the Consultation Centre of Psychotraumatology of the Social Pediatric Centre of the Cantonal Hospital Winterthur writes that: “successful school integration… is difficult in children with a trauma-related disorder and often externalizing and internalizing behavioral abnormalities are major challenges for teachers” (2021).

## 2 Trauma reactions in children and adolescents

In his work with traumatized children at the Children’s Hospital Zurich, Professor Markus Landolt was able to identify frequently occurring reactions in children and adolescents (2021). These behavioral problems can also be observed in children and adolescents with a refugee background. The reactions are grouped in Table 1 into those for the clinical picture of PTSD (post-traumatic stress disorder) according to DSM-5 (American Psychiatric Association, 2013; American Psychiatric Association, 2018).

**TABLE 1:** Diagnostic criteria according to DSM-5 (American Psychiatric Association, 2013; American Psychiatric Association, 2018) for post-traumatic stress disorder for adults, adolescents, and children, Landolt (2021).

Criterion A	Confrontation with actual or imminent death, serious injury or sexual violence in one or more of the following ways: A1: Direct experience of one (type I) or more (type II) traumatic events A2: Personal experience of traumatic events (type I or type II) in other people A3: Learn that a family member or close friend has experienced traumatic events (type I or type II). A4: Experience of repeated or extreme confrontation with aversive details of traumatic events (type I or type II) (e.g. first responder)
Criterion B	Intrusions B1: Recurrent, involuntarily imposing stressful memories of the traumatic events (type I or type II) B2: Recurring, distressing dreams that relate to the traumatic events (type I or type II) (in children also anxiety dreams without recognizable content) B3: Dissociative reactions (in children also “traumatic play”) B4: Intense or persistent psychological distress by internal and external cues of the trauma B5: Significant physical reaction to internal and external cues of the trauma
Criterion C	Avoidance C1: Avoidance of distressing memories, thoughts and feelings of the traumatic events (type I or type II) C2: Avoidance of environmental factors (people, places, conversations, activities, etc.) that are reminiscent of the traumatic events (type I or type II)
Criterion D	Negative change in cognition and mood D1: Inability to remember one or more important aspects of traumatic events (type I or type II) D2: Persistent and exaggerated negative beliefs or expectations that relate to one’s own person, other people, or the world D3: Persistent distorted cognitions regarding the cause and consequences of traumatic events (type I or type II), leading to attributing blame to oneself or others D4: Persistently negative emotional state D5: Significantly reduced interest or decreased participation in important activities D6: Feelings of separation or alienation D7: Persistent inability to feel positive emotions
Criterion E	Hyperarousal E1: Irritability and outbursts of anger E2: Risky and self-destructive behavior E3: Excessive vigilance E4: Exaggerated startle response E5: Difficulty concentrating E6: Sleep disorders
Criterion F	The disorder (criteria B, C, D, and E) lasts longer than 1 month
Criterion G	The disorder causes clinically significant suffering or impairment in social, occupational, or important functional areas
Criterion H	The disorder is not a consequence of the physiological effects of a substance
Determine whether	With dissociative symptoms 1: Depersonalization 2: Derealisation
Determine whether	With delayed onset

Against the background of these behavioral problems in traumatized children and adolescents, the School Psychological Service of the City of Zurich created the “group therapy for children and adolescents with traumatic experiences.” This project was pioneered by the couple Celi and Vicky Reiff (Paterson et al., 2016) who have been working with refugee children from the city’s schools for now almost 30 years, using creative methods with a depth-psychological and psychoanalytical basis (Rumpel et al., 2022). Our second trauma group therapy with karate-do for war-traumatized children and adolescents, which has now been offered in the city of Zurich for two years (Straub and Ortiz, 2023; Stadt Zürich Schul- und Sportdepartement, 2023), is designed with a new approach on the foundations of the concept of football group therapy of the Social Pediatric Center of the Cantonal Hospital Winterthur (Bamert et al., 2020).

## 3 Methods

The children get to know and apply methods of self-defense with the experienced karate-do instructor Patricio Ortiz. In group therapy, they learn to use the defense techniques of karate-do to real-life situations. At the same time, the experienced psychotherapist Mirjam Straub Ortiz (MSc) teaches the children how to handle their traumatic experiences with care. She draws on methods such as trauma-focused cognitive behavioral therapy (TF-CBT), Narrative Exposure Therapy (NET), and Integrative Gestalt Therapy according to Dr. Hilarion Petzold (EAG-FPI).

### 3.1 Methods of Karate-do and integrative budo-therapy

Budo-Terapy is understood against the background of the concept of “martial arts” by Professor H. Petzold, as: “Peace work with its healing potential, as a work of life design with the aim of personal sovereignty and art of living…” (2017).

Several articles (Ludwig, 2021; Siegele, 2013; Siegele, 2018) prove that, quote: “Integrative Budo-Therapy primarily through the attitude and teaching of social skills and abilities, as well as through the promotion of psychophysical regulatory skills, (makes) an important contribution to the… integration.” (Ludwig, 2021)

Budo includes the whole of the “martial arts”, - in our therapy approach we have specifically chosen the martial arts of Karate-do.

Karate is a specific physical activity which focuses on self-regulation and self-development; therefore, it reduces impulsivity and improve self-control.

The scientific study by * Potoczny et al. (2022) showed how Self-Control and Emotion Regulation of Karate training had an important mediating impact on subjective well-being (2022).

Karate training can therefore be used as an important intervention strategy in shaping volitional and personality characteristics - specifically in children and adolescents whose personality is still developing and can contribute to increasing their well-being (Supplementary Figure 1).

### 3.2 Methods of trauma-focused cognitive behavioral therapy (TF-CBT)

The trauma group therapy with karate-do for war-traumatized children and adolescents draws on trauma-focused cognitive behavioral therapy too (Cohen et al., 2009).

We chose to work with trauma-focused cognitive behavioral therapy for children and adolescents (TF-CBT) as there were showed large improvements across all outcomes from pre- to post-treatment in a systematic review carried out on 4523 minor participants from 28 RCTs and 33 uncontrolled studies which Thielemann et al. (2022). carried out in 2022. Effects were even more pronounced for group settings. So we considered to include this therapy approach for our group therapy.

This skill- and resource-based model builds on a sequence of components. The ones adopted for group therapy include the following items: psychoeducation (P), relaxation (R), affect regulation (A) and cognitive coping and processing (C), trauma narration (T):

*Psychoeducation* (P) is about providing children with knowledge about the traumatic experience, typical reactions and symptoms, triggers and treatment. Objective: The children can identify triggers and know adequate words to classify and describe the symptoms.

In *relaxation* (R), the children learn individualized relaxation skills (imagination techniques such as safe place, focused breathing from tai-chi, relaxation exercises from karate). Objective: The children can use apply relaxation skills in response to their PTSD symptoms.

In *affect regulation* (A), the children are provided with skills to express, recognize and regulate feelings. Objective: The children can regulate their feelings related to PTSD symptoms.

In contrast to TF-CBT, *cognitive coping and processing* (C) is adopted in our group therapy through self-control of one’s own body, using techniques from karate. Objective: The children and adolescents can perceive and control their own body and mind (self-control).

### 3.3 Narrative Exposure Therapy (NET)

In the “trauma narration” (T) part of the program, which is also used in the TF-CBT, we rely on the method of Narrative Exposure Therapy (NET) in the form of the Life-Line (Neuner et al., 2021). Since many traumatized children and adolescents in our group therapy can be assumed to suffer from “sequential traumatization” in the sense of Keilson and Sarphatie (1992), we have chosen as our approach to trauma exposure the form of a personal Life-Line and then a group life-line, to which all children and adolescents contribute and thus share one or more of their traumatic experiences in the group.

In the Life-Line, a biographical overview is interpreted spatially as an overall view in the form of a timeline on which lifetime periods, general and specific events are represented in a symbolical manner. Natural objects - such as flowers and stones - which symbolize positive and negative valence are often used to represent the events (Schauer et al., 2018).

In the group lifeline, a symbolic timeline is placed from the beginning of life to the present time on which each group member places a life event that is significant for him/her (usually a traumatic event is chosen) symbolized by a flower or a stone. In the following sequence a mutual “Sharing” i.e. Telling the group about the event takes place that deepens mutual trust and respect for the other group members.

### 3.4 Approaches of integrative gestalt therapy according to Hilarion petzold (EAG-FPI)−the tetradic system in integrative therapy

In his process model, Prof. Dr. mult. Hilarion Petzold has structurally combined the elements context/continuum, theme, and intersubjective constellation into a large whole (2003). The conception of his “Tetradic System” was influenced by considerations from other therapeutic phase and process models, such as Perls’ Gestalt therapy, Moreno’s psychodrama, and Iljine’s therapeutic theatre. From the comparison of the “remembering, repeating, working through” of Freud’s psychoanalysis, Lewin’s “unfreezing, change, refreezing,” and other dramatic, creative and problem-solving process approaches, Petzold developed the “dramatic curve” of his system (ibid., 2003). The ideal-typical course of a process comprises four phases in the “tetradic system” of integrative therapy: initial phase, action phase, integration phase, and reorientation phase (Leitner and Höfner, 2010).

In the trauma group therapy with karate-do for war-traumatized children and adolescents, the “tetradic system” according to Petzold is used to structure and thematically classify the process that the therapy undergoes. Since the context and the temporal continuum are often lost in the memory the traumatic event, this placement in space and time is of central importance in our group therapy.

#### 3.4.1 Initial phase: differentiation−complexity

“The initial phase first serves to *perceive* the situation, the context in which, with which and about which I want to co-respond. Perception brings me into *contact* with others. In contact, there is a *grasping* that goes beyond perception, I experience the other, the other experiences me, I experience myself through the and with the other, a “chance to encounter” arises” (Petzold, 2003).

In the trauma group therapy with karate-do for war-traumatized children and adolescents, this first phase consists of the process of group formation: A process of becoming aware of oneself (self-consciousness) through karate exercises as well as of the other(s) through joint training and play. Mutual respect and adherence to rules in the group are central to this stage.

Hanna Wintsch, a rennomated trauma psychotherapist in Switzerland and leading psychologist at the Eastern Swiss Children’s Hospital, has worked in war, post-war and crisis areas with children and adolescents. She developed her therapeutic group approach for children and adolescents locally in Bosnia in 1998, later tested, adapted and differentiated in Kosovo and Palestine under partly different framework conditions. Wintsch advocates that the building of trust should take place as the first prerequisite in a group therapy (initial phase), in order to create the fundament of a group so that in a second phase the traumatic experiences can be expressed and processed therapeutically (Wintsch, 2000).

Starting from this consistent initial phase, the themes of psychoeducation (P), relaxation (R), affect regulation (A), cognitive coping and processing (C), and trauma narration (T) are then worked on through the following phases: action (structuring−conciseness), integration (integration−stability), and finally reorientation (creation−transgression).

#### 3.4.2 Action phase: structuring−conciseness

The action phase is about a struggle for different approaches to solving the theme perceived and grasped in the initial phase: “The synergistic events of the action phase are characterized by such disputes between perceptions, concepts, persons, and subgroups and accumulate at the moment of highly synergistic conciseness, which opens up the dimension of understanding beyond perception and grasping: in consensus (which can also consist in the fact that there is dissent at the given time)” (Petzold, 2003).

In the trauma group therapy with karate-do for war-traumatized children and adolescents, the second phase seeks to deal with the theme: e.g. the theme of “trauma” and the resulting symptoms, the topic of “safety” and relaxation, or the theme of “affects” and the affective expression. The children and adolescents are encouraged in the group to comment on these themes and also to create conciseness in the joint discussion, which should lead to a better, deeper, as well as physically “embodied” understanding of the theme.

#### 3.4.3 Integration phase: integration−stability

The integration phase focuses on the task of critical evaluation: “The evaluation aims to critically emphasize the meaning of the event, its significance, to critically assess what has been achieved and to move on to consequences for action. The solution(s) or central aspects of the… theme are symbolically – usually linguistically – captured in a concise way” (Petzold, 2003, p. 130). This stage of the process is about understanding and explaining.

In the trauma group therapy with karate-do for war-traumatized children and adolescents, the “embodied” understanding that has now been developed is taken up again and expressed individually. This is done not only linguistically, but also symbolically with creative media (e.g. a body chart for the representation and location of feelings in the body). An attempt is made to enact the meaningfulness of the theme – in the context of one’s life.

#### 3.4.4 Reorientation phase: creation– transgression

“In the reorientation phase, the consensus is worked out in its consequences for action, as *preparation* for cooperative action (e.g. through simulation procedures, planning and role-playing, socio-drama, behavior drama) and subsequently through *transfer* to… the everyday situation” (Petzold, 2003).

In the trauma group therapy with karate-do for war-traumatized children and adolescents, an attempt is made to go move from understanding into action. The children and adolescents are encouraged – by means of certain skills learned in the group – to try out alternative ways of behavior in everyday (school) life.

## 4 Setting

The setting is embedded in the School Psychological Service of the City of Zurich and the back office: Teachers register children with the school psychologist, i.e., the specialist for and head of trauma (lic. phil. Catherine Paterson, School and Sports Department of the City of Zurich). The specialist’s management coordinates allocation to the group together with a secretary. Transport is also organized. Costs are covered by the school of the City of Zurich. Space is provided by the school. The therapy group accommodates up to fifteen 9–15 year old pupils from the city’s public schools. Every child has the right and the chance provided by the School Psychological Service of the City of Zurich to participate for 3 years in the group. We will soon have our first participants leaving us – due to the 3 years our program is running.

### 4.1 Length, duration and session structure of group therapy

We always make two parts in the one and a half hours in which we have group therapy every week: A first unit with Karate-do training, then we have a break where we provide a healthy snack to our participating children and then we continue with a second unit of psychoeducation and therapy on trauma topics.

## 5 Results

Our group has been running for two years, in which we have focused primarily on the components of psychoeducation, relaxation, affect regulation, and self-control. It has become apparent that the process of group building particularly, of establishing an atmosphere of confidence and for expressing feelings in a constructive way, needs intense therapeutic work. Having evaluated the interaction of the children with each other, we can see a change of behavior in self-reflection (sharing own thoughts and experiences), better recognizing and expressing feelings as well as improved abilities to listen to each other’s stories.

In 2021, we started working with a very heterogeneous group of children: Thy came from Iraq, Syria, Brazil, Peru, Eritrea and Somalia, among others. When the war in Ukraine started in 2022, many Ukrainian children came to the Swiss schools. Currently, we consider it useful to offer a separate group for these children with translation.

We offered back then both groups alternately every two weeks for 1,5 hours each.

Over time, it has been shown that there is a high fluctuation among Ukrainian families, and a large termination rate - the reason for which we do not yet know exactly. Also, the rhythm of group therapy only every two weeks seemed too little intense to us for the severity of the traumatization.

So in favor of better continuity the two groups were integrated for the school year 2023. They are now heterogenous about the country of origin, but we still have the privilege of a translation into Ukrainian for the possible language problems of Ukrainian children. Also, we now count up to 12 group members (before it was until 10).

## 6 Discussion

The therapy group is process-oriented, requiring constant reflection and evaluation. Working with traumatized children demands a highly sensitive perception of their needs in the moment and the ability to react in a prompt, flexible, and often creative way. Our trauma group therapy with karate-do for war-traumatized children and adolescents provides a nonverbal, embodied approach and is therefore offering different and more therapeutic possibilities. The group also creates the possibility for the children to be part of a group, which can represent a safe space and convey belonging.

Based on our behavioral observations and feedback from teachers, first experiences show positive results: We from the leading team of group therapy have the opportunity to exchange one hour per participating child with the teachers by phone about their progress over the year.

We have received good qualitative feedback from the teachers that it is now better possible for the children who go into therapy with us to concentrate at school and also to better regulate their feelings. We are also sometimes invited to meetings at school, where the child himself, his parents, the teachers and sometimes a person for translation are present to evaluate the general school success. These sessions are an opportunity for to explain to the parents in detail how we work with their children in therapy. And in most cases, we experience great sympathy and approval from them. It must not be forgotten that the children usually come from traumatized family relationships, where the parents themselfes are often tramatized and cannot help their children well with this topic. This makes the school context all the more important in which the trained teachers notice such tramatization and then refer the child - always with the consent of the parents - to our group therapy.

In 2023, we were also able to make a short documentary film for schooling purposes about our group therapy and showed it to the children, parents, grandparents and teachers recently in a gathering in July 2024 at the end of the school year: The feedback was extremely positive.

An initial scientific testing and then evaluation of our program is planned, but unfortunately not yet approved by the school.

## Data Availability

The original contributions presented in the study are included in the article/Supplementary material, further inquiries can be directed to the corresponding author.

## Supplementary material

Supplementary Figure 1 Karate training in Trauma group therapy for war-traumatized children and adolescents.
