# Supplementary figures and images for: Case Report: Trauma group therapy with karate-do for war-traumatized children and adolescents
Source: Front Psychol. 2024 Oct 16;15:1301671. doi: 10.3389/fpsyg.2024.1301671 (PMC11523532; doi:10.3389/fpsyg.2024.1301671)

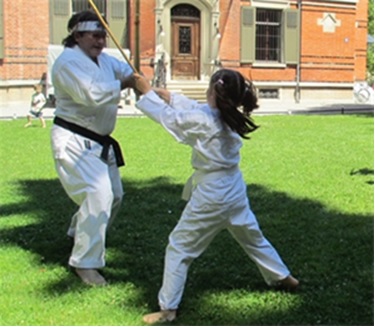

Supplement: Supplementary Figure 1 — Karate training in Trauma group therapy for war-traumatized children and adolescents. [file Image_1.JPEG]
